# Differential Modulation of Mouse Intestinal Organoids with Fecal Luminal Factors from Obese, Allergic, Asthmatic Children

**DOI:** 10.3390/ijms25020866

**Published:** 2024-01-10

**Authors:** Samir Córdova, Mireia Tena-Garitaonaindia, Ana Isabel Álvarez-Mercado, Reyes Gámez-Belmonte, Mª Amelia Gómez-Llorente, Fermín Sánchez de Medina, Ana Martínez-Cañavate, Olga Martínez-Augustin, Carolina Gómez-Llorente

**Affiliations:** 1 Departamento de Bioquímica y Biología Molecular II, Facultad de Farmacia, Campus de Cartuja s/n, Universidad de Granada, 18071 Granada, Spain; clamirja25@correo.ugr.es (S.C.); mireiatena@ugr.es (M.T.-G.); alvarezmercado@ugr.es (A.I.Á.-M.); gomezll@ugr.es (C.G.-L.); 2 Ibs.GRANADA, 18012 Granada, Spain; mariaa.gomez.sspa@juntadeandalucia.es (M.A.G.-L.); fsanchez@ugr.es (F.S.d.M.); 3 Departamento de Farmacología, Facultad de Farmacia, Universidad de Granada, 18071 Granada, Spain; mariadelosreyes.gamezbelmonte@uk-erlangen.de; 4 Unidad de Pediatría, Hospital Materno-Infantil, 18071 Granada, Spain; anamartinezcanavate@gmail.com; 5 Centro de Investigación Biomédica en Red-Enfermedades Hepáticas y Digestivas (CIBERehd), Spain; 6 Instituto de Nutrición y Tecnología de los Alimento José Mataix, 18071 Granada, Spain; 7 Centro de Investigación Biomédica en Red-Obesidad (CIBERobn), Spain

**Keywords:** organoids, fecal content, gastrointestinal microbiome, asthma, obesity

## Abstract

Asthma is a multifactorial condition that can be associated with obesity. The phenotypes of asthma in lean and obese patients are different, with proinflammatory signatures being further elevated in the latter. Both obesity and asthma are associated with alterations in intestinal barrier function and immunity, and with the composition of the intestinal microbiota and food consumption. In this study, we aimed to establish an organoid model to test the hypothesis that the intestinal content of lean and obese, allergic, asthmatic children differentially regulates epithelial intestinal gene expression. A model of mouse jejunum intestinal organoids was used. A group of healthy, normal-weight children was used as a control. The intestinal content of asthmatic obese children differentially induced the expression of inflammatory and mitochondrial response genes (*Tnf*-tumor necrosis factor, *Cd14*, *Muc13*-mucin 13, *Tff2*-Trefoil factor 2 and *Tff3*, *Cldn1*-claudin 1 and *5*, *Reg3g*-regenerating family member 3 gamma, *mt-Nd1*-NADH dehydrogenase 1 and *6*, *and mt-Cyb*-mitochondrial cytochrome b) via the RAGE-advanced glycosylation end product-specific receptor, NF-κB-nuclear factor kappa b and AKT kinase signal transduction pathways. Fecal homogenates from asthmatic normal-weight and obese children induce a differential phenotype in intestinal organoids, in which the presence of obesity plays a major role.

## 1. Introduction

Asthma is a chronic inflammatory disorder characterized by a reversible airflow obstruction, with airway inflammation playing a central role in its pathogenesis [1,2]. Different underlying molecular mechanisms define asthma endotypes, which are linked to different phenotypes. In this regard, obesity has been found to be a distinguishing variable in the classification of asthma subtypes [3,4]. Obese asthmatic patients are prone to corticoid resistance and have a higher risk of being hospitalized [5,6]. Asthma and obesity are both considered chronic inflammatory disorders, and the features of inflammation in these conditions are enhanced when combined [4]. The risk and severity of asthma and obesity have been associated with alterations in immunity and the intestinal microbiome, and therefore, with alterations in the mucosal barrier’s function [7]. In fact, we have previously characterized the molecular heterogeneity of the allergic asthma phenotype in children (BIOASMA cohort) using a multi-omic approach combining clinical data, plasma and fecal inflammatory biomarkers, metagenomics and metabolomics [8]. In a similar way, Michalovich et al. showed changes in intestinal microbiota composition in asthmatic and obese patients that were increased in patients with both conditions and correlated with inflammatory markers in serum [4]. Additional effects on mucosal barrier disruption have also been observed in animal models that combine both conditions [9].

It is increasingly believed that diet and an altered gut microbiota may play an important role in the pathology of asthma and obesity. The intestinal epithelium is a cell monolayer with an essential role in the mucosal barrier’s function, being the first line of defense against pathogenic bacteria [10]. It also interacts with luminal food components and commensal bacteria that, in turn, shape intestinal function, including the intestinal epithelium itself. Thus, the intestinal epithelial’s immune response, together with its epithelial permeability or proliferation and differentiation rates, are regulated by luminal contents. Gut metabolites jointly reflect diet intake, modified human metabolites, microbial metabolism and environmental chemical compounds, all of which shape the barrier’s function [11].

As the long-term culture of intestinal epithelial is generally not possible, up until a few years ago intestinal cell Iines were the only in vitro intestinal models available for testing the effects of molecules, including nutrients. Nevertheless, cultured cell lines, as a model, are very limited because of their immortalized nature and the lack of different cell types present in the intestinal epithelium [12]. Organoids, obtained from intestinal stem cells, contain all epithelial cell lines and overcome these limitations, being a much more physiological model. They can be obtained from any intestinal segment and from several species. As an available human tissue is needed to establish human organoids, it is quite difficult to obtain small intestinal organoids of human origin. Hence, we planned to use a model of mouse organoids, avoiding the use of invasive techniques in human beings, as a physiologically relevant model to study disease-dependent dysfunction in the intestinal epithelial barrier. Therefore, in the present study, we aim to ascertain whether the intestinal content of children with asthma alone or in combination with obesity (BIOASMA cohort) differentially regulates the intestinal epithelium using a model of mouse jejunum organoids.

Fecal homogenates from normal weight and obese asthmatic children were added to a cell culture medium and the expression of intestinal barrier, immune and mitochondrial respiratory chain genes were studied. Fecal homogenates from normal weight healthy children were used as a control.

## 2. Results

### 2.1. Cohort Description

Normal weight and obese asthmatic prepubertal children from the BIOASMA cohort [8] were included in our study. The clinical characteristics of these children are shown in Table 1, together with plasma determinations and fecal D-lactate concentrations, for reference. Simultaneous T-test analysis followed by a correction of multiple comparisons using the Holm–Sidak method showed that, as expected, BMI (Body mass index) and BMIZ (Age and sex standardized body mass index) scores were higher in asthmatic obese (AO) children. Leptin concentration in the plasma of obese children was also higher than in that of normal weight participants. No differences were observed in other measured parameters (Table 1).

### 2.2. Fecal Waters from Overweight but Not Normal-Weight Allergic–Asthmatic Children Induce the Expression of Proinflammatory and Intestinal Barrier Function Genes

**Figure 1:**
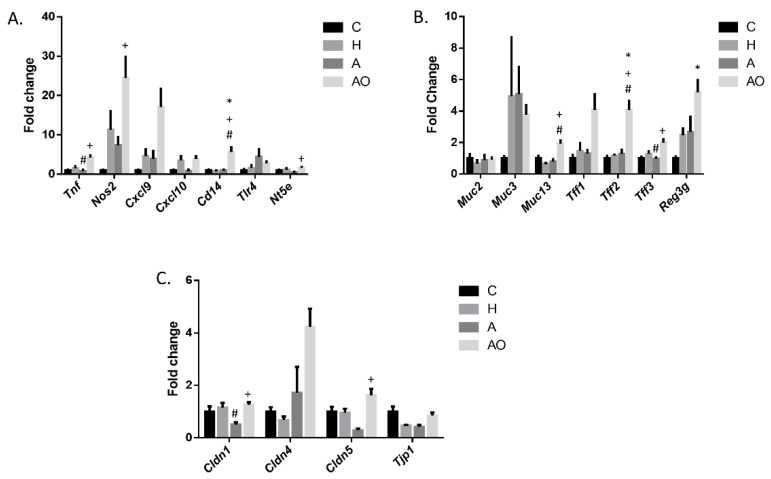
Effect of fecal homogenates of children on the expression of intestinal barrier function-related genes. (**A**) Inflammation-related genes. (**B**) Mucins, Trefoil factors and *Reg3g*. (**C**) Permeability-related genes. Fecal homogenates from healthy (H), normal-weight asthmatic (A) and obese asthmatic (AO) children were added to the cell culture medium of mouse jejunum organoids. qRT-PCR was performed. All the samples were separately assayed. Results are expressed as mean ± SEM fold change vs. control medium (C). One-way ANOVA followed by Tukey test were applied. ^+^: *p* < 0.05 vs. A; ^#^: *p* < 0.05 vs. H; *: *p* < 0.05 vs. C.

Changes in the intestinal barrier’s function and inflammation have been described in asthma and obesity. Our first goal was to study whether the intestinal content of A and AO children was able to modulate the expression of genes that regulate these functions. To this end, we added fecal homogenates to the cell culture medium of mouse jejunum organoids. For this analysis we also included feces from a group of prepubertal healthy children (H). Our data indicate that *Tnf* (Tumor necrosis factor) and *Nos2* (Nitric oxide synthase 2), traditional markers of inflammation, were induced in mouse jejunum organoids after the addition of homogenates from AO children when compared to the addition of A homogenates (Figure 1A). The same effect was observed for *Nt5e*, which codifies an ectonucleotidase, and CD73, which plays a major role in the local production of anti-inflammatory adenosine. *Cd14*, a co-receptor of TLR4 which recognizes pathogen-associated molecular patterns, was also significantly stimulated by AO homogenates when compared to all the other groups assayed. *Cxcl9* and *Cxcl10* are chemokines which are important in antibacterial defense. The ANOVA p value for *Cxcl9*, with a fold change of 4.33 compared with the H group, indicated a tendency for significance (*p* = 0.061). In turn, A homogenates only produced a decrease in *Tnf* expression when compared to H homogenates, and no effect of H homogenates was observed (Figure 1A).

Mucins are a key component in intestinal mucus, protecting the epithelium from bacterial invasion. Together with trefoil factors (TFFs), which are mucin-associated secretory molecules, mucins contribute to the intestinal barrier’s function and are mainly produced by goblet cells. The expression of *Muc13* and genes encoding TFF2 and 3 (*Tff2* and *Tff3*) was induced by the addition of fecal waters from AO children to the culture media of mouse jejunum organoids (Figure 1B). A similar pattern was observed for *Reg3g* (Regenerating family member 3 gamma), whose expression was statistically induced when compared to that of the control (C) organoids. This gene codifies a lectin that is an important component of the intestinal barrier, maintaining the segregation of bacteria from the host. Although there are other Reg3 gamma proteins, Reg3γ is the homolog for human REG3A, which is the one expressed at the highest levels in the human small intestine [13]. No effects of A or H homogenates were observed, except for an inhibition of *Tff3* in the A group when compared to the H group (Figure 1B).

The maintenance of physiological intestinal permeability is essential for intestinal barrier function. Alterations in this permeability have been related to both asthma and obesity. Claudin 1, 4 and 5, and zonulae occludens-1 (ZO-1), are part of the tight junctions proteins that regulate intestinal permeability. A study of the expression of genes that codify these proteins indicated that *Cldn1* and *Cldn5* were increased in the AO group when compared to the A group (Figure 1C). In turn, a decrease in *Cldn1* expression in A vs. H was observed. ANOVA analysis of *Cldn4* and *Tjp1* (which codifies ZO-1) indicated no specific differences between the groups, although the ANOVA p value was 0.011 in the first case, and pairwise T-tests between C and AO and H and AO were significant.

**Figure 2:**
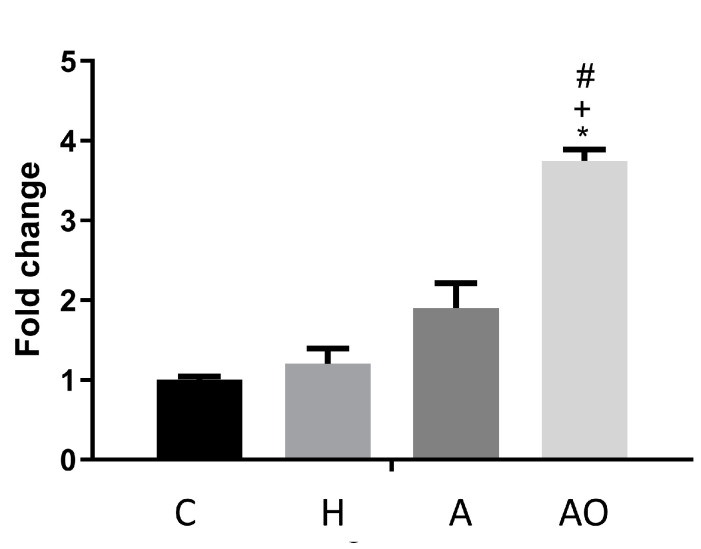
Effect of fecal homogenates of children on the expression of *Cnr1*. Fecal homogenates from healthy (H), normal-weight asthmatic (A) and obese asthmatic (AO) children were added to the cell culture medium of mouse jejunum organoids. Three pools of n = 3 and n = 6–7 samples were assayed for the A and AO groups, respectively. qRT-PCR was performed. Results are expressed as mean ± SEM fold change vs. control medium (C). One-way ANOVA followed by Tukey test were applied. ^+^: *p* < 0.05 vs. A; ^#^: *p* < 0.05 vs. H; *: *p* < 0.05 vs. C.

*Cnr1* encodes the cannabinoid receptor 1, whose expression has been shown to be induced in inflammatory conditions and seems to be related to the regulation of intestinal permeability when a high-fat diet is administered [14]. The expression of *Cnr1* was induced by the homogenate of feces from AO children when compared to the other study groups (Figure 2).

**Figure 3:**
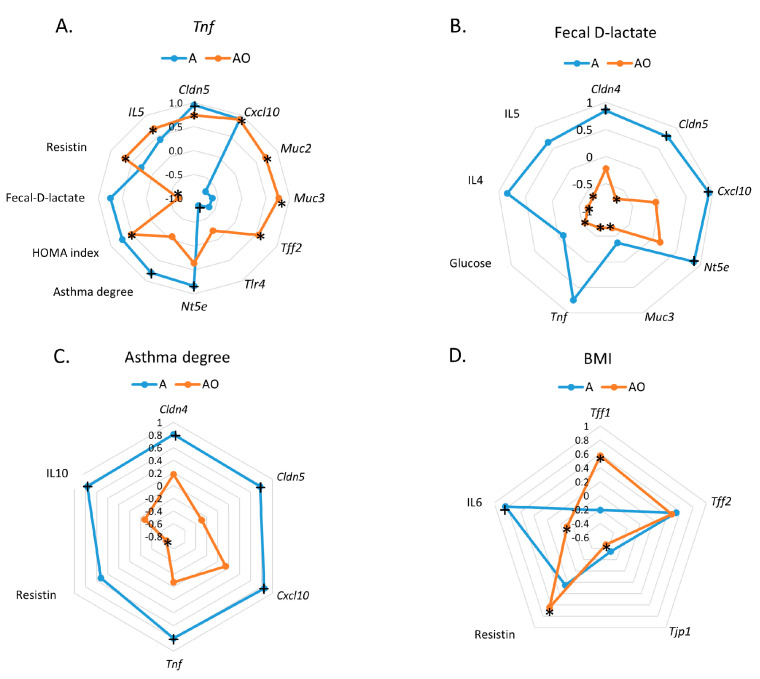
Radial charts for statistically significant correlations between TNF (**A**), fecal D-lactate (**B**), Asthma (**C**) and BMI (**D**) and parameters assayed in normal-weight asthmatic (A) and obese asthmatic (AO) prepubertal children. Pearson correlation coefficients with correction for multiple comparisons using the Holm–Sidak method are shown. +: *p* < 0.05 for correlation coefficients in the A group *: *p* < 0.05 for correlations coefficients in the AO gruop.

Correlations between measured parameters in intestinal organoids were studied to further characterize the differential effect of the intestinal content from A and AO children (Table 2 and Table 3). Multiple correlations were found for both groups in this analysis, showing the strong relationship between the parameters studied. All significant correlations found between parameters in the AO group were positive (Table 3), while some negative correlations were found in the A group (Table 2). Thus, *Tff2* and *Tff3* were negatively correlated with *Muc13*. *Tff3* also was negatively correlated with *Cldn4*, *Cldn5* and *Cxcl10*. Finally, a negative correlation in the A group was also found between *Tlr4* and *Cldn5* and between *Tnf* and *Tlr4*. *Tnf* is one of the most relevant molecules involved in intestinal inflammation. Figure 3A shows radial charts for the correlations found for *Tnf* in order to illustrate the differences between the A and AO groups. Both *Cldn5* and *Cxcl10* were positively and strongly correlated with *Tnf* in both groups, while the barrier function parameters (*Muc2*, *Muc3* and *Tff2*) were only positively correlated with *Tnf* in the AO group. Correlations with *Tlr4* (negative) and *Nt5e* (positive) were found in the A group and not in the AO group. No correlations were found with any parameters in the A group (Figure 3D).

Fecal D-lactate originates from intestinal bacteria in the intestine. Although the levels measured in both groups were very similar, the correlations found with parameters determined in organoids were very different. A highly significant positive correlation was found for *Cldn4* and *Cldn5,* and *Cxcl10* and *Nt5e* in the AO group, while negative correlations were found for *Cldn5*, *Muc3* and *Tnf* in the A group (Figure 3B).

To further establish the relationship between allergic asthma associated with obesity and the parameters of intestinal barrier function and inflammation, we used correlation analyses. Correlations of *Tnf* with systemic parameters further indicated differences between both groups. Thus, while in group A, *Tnf* was positively correlated with the asthma degree, in the AO group, it positively correlated with the HOMA (Homeostasis model assessment) index and resistin, indicating that, in the case of AO, changes in feces induced by obesity are more important than those promoted by allergies (Figure 3A). This hypothesis was reinforced by the fact that asthma degree and BMI were correlated with distinct parameters in the A and the AO groups, respectively (Figure 3C,D). Thus, a positive correlation of asthma with systemic IL-10 and organoid expression of *Tnf*, *Cxcl10*, *Cldn5*, *Cldn4* in the A group was found (Figure 3C). In contrast, no significant correlations were observed between asthma degree and any of these parameters in the AO group. In turn, BMI correlated positively with *Tff1* and *Tff2* and negatively with *Tjp1* in the AO group, while no correlations were found with any parameters in the A group (Figure 3D).

### 2.3. RAGE, IP3K and NFκB Mediate the Induction of the Expression of Intestinal Barrier Function-Related Genes by Fecal Waters of Obese Allergic Children

**Figure 4:**
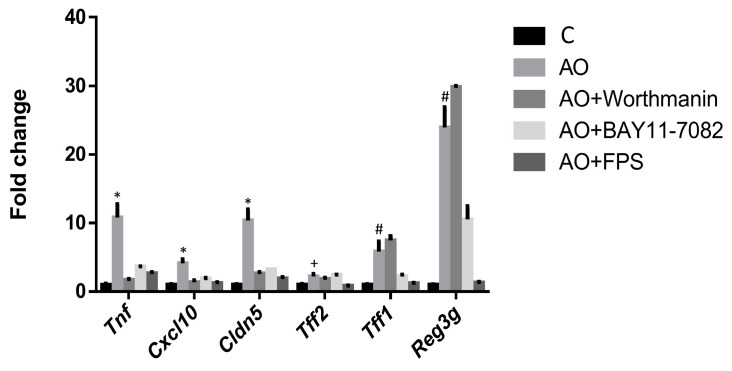
Effect of signal transduction inhibitors. Fecal homogenates from obese asthmatic (AO) children were added to the cell culture medium of mouse jejunum organoids together with inhibitors of AKT (wortmannin), NFκB (BAY11-7082) and RAGE (FPS-ZM1) signal transduction pathways. qRT-PCR was performed. Results are expressed as mean ± SEM fold change vs. control medium (C). One-way ANOVA followed by Dunnett’s multiple comparisons tests was applied to study differences with the AO group. *: *p* < 0.05 vs. all; ^#^: *p* < 0.05 vs. all except AO + wortmannin; ^+^: *p* < 0.05 vs C and AO + FPS.

The involvement of three main signal transduction elements (NFκB, IP3K and RAGE) in the effect of the fecal waters from AO children on intestinal gene expression was assessed next. Our results indicate that the studied elements are important in the observed effects, with RAGE apparently being a common mediator in the response of the assessed genes (Figure 4). In fact, the addition of the RAGE inhibitor N-benzyl-4-chloro-N-cycohexylbenzamid (FPS-ZM1) to the culture media of organoids before the AO fecal homogenates abrogated their effect on the expression of all the genes studied (*Tnf*, *Cxcl10*, *Cldn5*, *Tff2*, *Tff1* and *Reg3g*). The NFκB inhibitor BAY11-7082 also strongly abrogated the response elicited by AO fecal homogenates in all these genes, except *Tff2*. Finally, wortmannin, the IP3K kinase inhibitor, prevented *Tnf*, *Cxcl10* and *Cldn5* responses.

### 2.4. Fecal Waters from Obese Allergic Children Induce the Expression of Mitochondrial Respiratory Chain Genes

**Figure 5:**
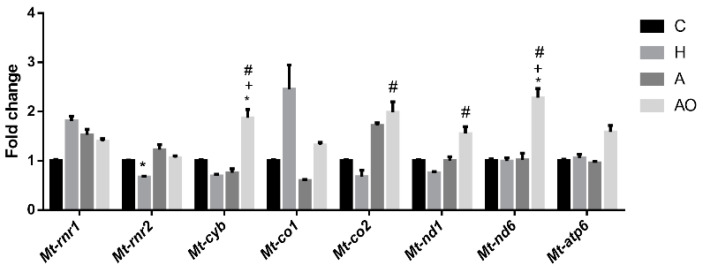
Effect of fecal homogenates of children on the expression of mitochondria-related genes. Fecal homogenates from healthy (H), normal-weight asthmatic (A) and obese asthmatic (AO) children were added to the cell culture medium of mouse jejunum organoids. Three pools of n = 3 and n = 6–7 samples were assayed for the A and AO groups, respectively. qRT-PCR was performed. Results are expressed as mean ± SEM of fold change vs. control medium (C). One-way ANOVA followed by Tukey test was applied. ^+^: *p* < 0.05 vs. A; ^#^: *p* < 0.05 vs. H; *: *p* < 0.05 vs. C.

Obesity, and its associated inflammatory process, may lead to an increased production of reactive oxygen species and cause oxidative stress. Moreover, excess nutrient supply can overwhelm the Krebs cycle and the mitochondrial respiratory chain, causing mitochondrial dysfunction that, in turn, increases the production of reactive oxygen species (ROS) and exacerbates the inflammatory process. Since a proinflammatory effect was observed for AO fecal waters, we aimed to study their effect on the expression of genes in the mitochondrial respiratory chain (Figure 5). The expression of respiratory chain genes from complexes I (*mt-Nd1 and 6*), III (*mt-Cyb*) and IV (*mt-Co1 and* 2) was assessed. In addition, *mt-Atp6* and mitochondrial 12S and 16 rRNA genes (*mt-Rnr1* and *mt-Rnr2*) were studied. AO fecal waters induced the expression of genes from complexes I and III and of *mt-Co2*. *mt-Atp6* was not significantly induced (the ANOVA *p*-value was 0.07). Neither were the mitochondrial rRNA genes. No effects of A or H fecal waters were observed, except for the inhibition of the expression of *mt-Rnr* in the H group.

## 3. Discussion

Asthma and obesity are both related to an altered microbiota and intestinal barrier, thus, the combination of obesity and allergic asthma has been associated with more severe symptoms, a higher frequency of exacerbation episodes and a lower response to therapy [15]. Although the implication of the intestinal microbiota in both pathologies is well known, the pathophysiological relationship between the fecal content and intestinal function is not fully understood. In this study we have determined how the intestinal content of obese and normal-weight prepubertal allergic-asthmatic children can alter intestinal function. For that, we have used a model with mouse jejunal organoids that were cultured with fecal homogenates from these children, in order to characterize differences in the expression of genes which are relevant to immunological and intestinal barrier function. It is interesting to note that fecal homogenates were obtained after sonication and filtration, therefore no bacteria were present, only bacterial components and metabolites were included.

As previously described, the clinical characterization of our cohort revealed that obese asthmatic children presented not only a higher BMI, but also increased plasma leptin levels [8]. This hormone, produced by white adipose tissue, has been observed to be augmented in obese individuals, who develop resistance to the hormone, and as a consequence, a reduction in the feeling of satiety, leading to greater energy consumption. It has been described that, in obese asthmatic individuals, increased leptin levels could modify airway function and result in greater inflammation [16,17]. However, no other differences were found in plasma parameters in our cohort, indicating generally similar levels of asthma and systemic inflammation in both groups. We are aware that the sample size could be a limitation of our study, but this is mitigated by the substantial phenotypic differences originally observed in the cohort, which have also resulted in a differential profile of organoids.

Immunity, the mucus layer and the production of antibacterial peptides are important elements in the maintenance of intestinal barrier function [10]. In addition, adequate control of intestinal permeability is essential for the correct functioning of this barrier. Our results indicate that the feces of AO children, but not those of A children, induce the expression of inflammatory and intestinal barrier function genes. Specifically, in AO children-derived fecal waters, an increase in genes related to inflammation was observed, such as *Tnf*, *Nos2*, *Cd14*, *Nt5e* or *Cnr1*. Of note, the induction of inflammatory markers was accompanied by changes in genes associated with barrier function that, in general, tend to increase, perhaps to possibly counteract inflammatory effects. Thus, *Tnf* induction was, for example, positively related to the expression of genes related to intestinal barrier function (*Cldn5*, *Muc3*, *Muc3* or *Tff2*) globally. Notably, barely any effect on gene expression was noted for fecal waters of healthy or nonobese asthmatic patients. In this regard, it is worth noting that our model made use of intact spheric organoids in which the luminal side of the epithelium faces the inner cavity. Therefore, in our study the homogenates were in contact with the basal side. It should be noted that the passage of 4 kDa, and even 10 kDa, molecules from the outside to the luminal side of organoids has been shown [18]. Therefore, at least small molecules would be in contact with this side of the organoids. In addition, even if large molecules are in contact only with the basal side, we consider this model valid since intestinal permeability has been shown to be altered in both obesity and asthma, and therefore the contact of bigger molecules with the basal side of the epithelium would take place in these diseases. This said, it is possible that this lack of effect of the bacterial homogenates of asthmatic, normal-weight and of non-asthmatic children could be due to the fact that large molecules do not have access to the luminal side.

At any rate, our results delineate a differential effect related to the microbiota or the fecal metabolites of these patients. Minimal differences in microbiota composition were previously found in our cohort, pointing to the probable pivotal role of the metabolite profile, which is currently under characterization. Accordingly, in future research we plan to delineate the effect of bacterial homogenates using open organoids (monolayers). Nevertheless, although obtaining open organoids is possible, in our experience it is a quite inefficient procedure that so far makes their use difficult in studies with many samples.

D-lactate is produced by intestinal bacteria and has been proposed as a marker of microbiota imbalance. Although no differences were found in the levels of this metabolite in the feces of obese and normal-weight asthmatic patients, our data indicate that, in asthma patients, they are directly correlated with the expression of inflammatory markers (*Cxcl10*, *Nt5e*, *Cldn4* and *Cldn5*). In contrast, D-lactate negatively correlated with *Muc3*, *Tnf* and *Cldn5* in obese asthmatic children. The above-mentioned data, together with the fact that the inhibition of RAGE, NFκB and AKT prevented the overexpression of genes in obese asthmatic fecal waters, indicate that different molecular pathways are activated in normal weight vs. obese children with asthma. RAGE was apparently a common mediator in the response of the assessed genes. This receptor is a mediator of intestinal inflammation. In fact, *Rage*^−/−^ mice are less susceptible to developing intestinal and colonic inflammation than WT mice [13]. In addition, this is a receptor with a wide range of inflammation-related ligands, including, besides advanced glycation products, bacterial lipopolysaccharide or S100Ca^2+^-binding protein B (S100B) and calprotectin (S100A8/S100A9). The ablation of the inflammatory response after the addition of a RAGE inhibitor would be consistent with the proinflammatory effect of the molecules present in the feces of obese children.

Obesity leads to various changes in the body. Among them, the existing inflammatory process may increase the production of ROS and cause oxidative stress. Oxidative stress, in turn, can trigger mitochondrial dysfunction. It has been shown that high-fat diet consumption provokes a metabolic shift toward fatty acid β-oxidation in the small intestinal epithelial cells and impairs colonocyte mitochondrial function, possibly through the downstream consequences of excessive fatty acid β-oxidation and/or the presence of deleterious metabolites produced by the gut microbiota [13]. Our data indicate an induction of the expression of mitochondrial oxidative phosphorylation pathway genes in organoids after the addition of fecal homogenates from AO children, but again, not those from lean asthmatic children. These alterations may constitute an adaptation in the mitochondria to allow a higher energy intake that would lead to their increased proliferation but also to an elevated ATP synthesis and, as a consequence, to a higher production of ROS [19].

Our data indicate that obesity is a more important factor than asthma in inducing intestinal inflammation and alterations in intestinal barrier function. This hypothesis is supported by the above-mentioned data and by the fact that the correlations between parameters assessed in organoids differentially correlate with the degree of asthma and with the BMI in the group of normal-weight and overweight asthmatics. In the case of normal-weight children, where inflammation markers were not found to be elevated, positive correlations were observed between asthma severity and inflammation parameters (*Tnf*, *Cxcl10*, *Cldn4* and *Cldn5*), while no correlations were found for obese children. In whom, in turn, BMI was positively correlated with *Tff1* and *Tff2* and negatively with *Tjp1*. *Tjp1* encodes ZO-1, which is a scaffold protein which cross-links and anchors tight junction strand proteins regulating intestinal permeability and has been shown to be altered in obesity [20]. Further research will be needed, but these data suggest that barrier function may be modulated by luminal contents in obesity.

The model of intestinal organoids, as with every model, presents limitations, in this case working with closed 3D structures with limited access to the luminal side, but it has proven to be adequate for our purpose, since valuable information has been obtained.

## 4. Material and Methods

### 4.1. Patients

Our cohort included normal-weight, healthy children (group H, n = 4), and asthmatic–allergic children (n = 28) with several degrees of asthma and different body mass indexes (BMIs). Among the asthmatic–allergic children, 9 were classified as normal-weight (group A) and 19 as obese children (group AO) according to Cole et al. [6]. Regarding the severity of asthma, 5 were classified as occasional, 13 as frequent and 10 as persistent according to the Spanish Guidelines for Asthma Management (GEMA). The main clinical and anthropometric parameters, fecal and serum inflammatory biomarkers, metabolomic and metagenomic data of the asthmatic cohort are described in Gómez-Llorente MA et al. [8]. This former cohort (BIOASMA) included a higher number of children, but here we have studied those with available feces. Heathy children were recruited for this study. Criteria for inclusion were healthy prepubertal children with no asthma or other chronic conditions.

### 4.2. Organoid Experiments

#### 4.2.1. Fecal Water Preparation

Stool from children (groups H, A and AO) was weighed and mixed (1 g/mL) in a buffer containing phosphate-buffered saline (PBS), 20% fetal bovine serum (FBS) (Sigma-Aldrich, San Luis, MO, USA) (*v*/*v*) and 2 mM ethylenediaminetetraacetic acid (EDTA, Sigma-Aldrich). Fecal samples were centrifuged (24,100× g, 10 min). Then, the supernatant was sonicated at 50% amplitude for 10 s in an UP200S ultrasonic device (Hielscher, Berlin, Germany), centrifuged (24,100× *g*, 5 min) and filter sterilized (0.22 μm). This preparation was referred to as fecal water.

#### 4.2.2. Crypt Isolation and Organoid Culture from Mouse

Jejunum intestinal organoids were obtained as previously described [21]. Briefly, jejunum intestinal organoids from wild-type (WT) mice were dissected and incubated for 30 min at 4 °C in PBS with 2 mM EDTA. After shaking, dissociated fragments were passed through 70 µm filter and crypts were counted and centrifuged for seeding. The pellet was resuspended in Corning-Matrigel^®^ (Fisher Scientific, Madrid, Spain) and IntestiCult^TM^ (StemCell, Grenoble, France) with a 1:1 ratio. Domes were cultured in 24 well plates with IntestiCult^TM^ supplemented with penicillin–streptomycin (Sigma-Aldrich). Intestinal organoids were stimulated with 1% (*v*/*v*) of fecal water overnight and then processed for RNA extraction. For experiments with inhibitors, 10 µM of BAY11-7082, 10 µM FPS-ZM1 or 1 µM wortmannin (Sigma Aldrich) were added for 24 h and then RNA was extracted as previously described [22,23].

#### 4.2.3. Analysis of Gene Expression by RT-qPCR

Total RNA was isolated using the RNeasy Mini Kit (Qiagen, Barcelona, Spain). One mg was retro-transcribed, and specific RNA sequences were amplified with a Bio-Rad CFX Connect real-time PCR device (Bio-Rad Hercules, CA, USA). The 2^−ΔΔCT^ method was used for relative quantification using *18s* and *Hprt* (*Hypoxantine phosphoribosyltransferase*) as reference genes. Table 4 and Table 5 detail the primers used for qPCR analysis.

### 4.3. Data and Statistical Analysis

Results are expressed as mean ± SEM. Differences among means were tested for statistical significance using a one-way ANOVA followed by a Tukey or a Dunnett’s multiple comparisons test. Pearson correlation coefficient and the Holm–Sidak method were used to study correlations and to correct for multiple comparisons. All analyses were carried out with the GraphPad Prism 6 (La Jolla, CA, USA). Differences were considered significant at *p* < 0.05.

## 5. Conclusions

Taken together, our data indicate that feces from obese asthmatic children induce changes in intestinal epithelial cells, including mitochondrial dysfunction, increased inflammation and alterations in the intestinal barrier function, which are mediated by different signal transduction pathways. Our results also indicate that the model used is valid for detecting differences in gene expression brought about by fecal homogenates.

Fecal homogenates from asthmatic, normal-weight and obese children induced a different phenotype in intestinal organoids, in which the presence of obesity plays a major role. These effects may underlie the well-established increased severity of symptoms observed in obese asthmatic children.

## Figures and Tables

**Table 1:** Fecal D-Lactate, macroscopic parameters and plasma parameters in normal-weight asthmatic (A) and asthmatic–obese (AO) children. Mean ± SEM. A simultaneous T-test was performed, and *p* values are shown. *: significant after correction for multiple comparisons using the Holm–Sidak method. Data have been obtained from Gomez-Llorente MA et al. [8].

	A	AO	*p* Value
Asthma	1.889 ± 0.200	2.316 ± 0.172	0.148
Age	9.444 ± 0.884	8.474 ± 0.486	0.306
Weight (Kg)	31.122 ± 1.980	44.887 ± 2.886	0.005
Height (m)	1.383 ± 0.036	1.345 ± 0.028	0.426
BMI	16.156 ± 0.452	24.363 ± 0.601 *	<0.0001
BMIZscore	−0.489 ± 0.153	2.858 ± 0.160 *	<0.0001
Hip perimeter (cm)	70.714 ± 2.998	83.194 ± 2.482	0.010
Waist–hip ratio	0.837 ± 0.019	0.862 ± 0.023	0.487
Fecal D-Lactate (mM)	9.100 ± 1.329	10.327 ± 1.508	0.609
Glucose (mmol/L)	4.722 ± 0.224	4.629 ± 0.087	0.4439
Insulin (µUL/mL)	6.847 ± 1.886	8.606 ± 1.137	0.1759
HOMA index	1.144 ± 0.323	1.654 ± 0.169	0.585
LBP (µg/mL)	3.171 ± 0.604	3.600 ± 0.461	0.606
Adiponectin (mg/L)	7.071 ± 1.718	5.631 ± 0.726	0.378
Resistin (µg/mL)	13.671 ± 1.765	13.671 ± 1.133	>0.9999
Adiponectin/resistin ratio	0.400 ± 0.086	0.446 ± 0.071	0.706
Leptin (µg/L)	2.586 ± 0.440	8.156 ± 0.555 *	<0.0001
TNF (pg/mL)	3.800 ± 0.479	3.383 ± 0.229	0.385
IL-10 (pg/mL)	3.371 ± 0.341	4.114 ± 0.426	0.270
IL-13 (pg/mL)	2.517 ± 0.681	1.927 ± 3.373	0.419
IL-4 (pg/mL)	13.917 ± 3.200	12.846 ± 2.075	0.777
IL-5 (pg/mL)	1.200 ± 0.145	1.421 ± 0.114	0.263
IL-6 (pg/mL)	0.950 ± 0.228	1.099 ± 0.220	0.683
IL-8 (pg/mL)	1.957 ± 0.188	1.793 ± 0.212	0.624

**Table 2:** Correlations between parameters measured in intestinal organoids after the addition of feces from normal-weight asthmatic children. A higher-stringency Pearson correlation coefficient, calculated with all parameters in the study, is shown. *: *p* < 0.05.

	*Cd14*	*Cldn1*	*Cldn4*	*Cldn5*	*Cxcl10*	*Cxcl9*	*Muc2*	*Muc3*	*Muc13*	*Nos2*	*Nt5e*	*Tff1*	*Tff2*	*Tff3*	*Tjp1*	*Tnf*	*Tlr4*
*Cd14*		0.573	0.758 *	0.495	0.560	0.422	0.096	−0.132	0.867 *	−0.335	0.650	0.101	−0.556	−0.706	0.372	0.330	−0.474
*Cldn1*	0.573		0.922 *	0.466	0.738	0.609	0.234	0.408	0.213	−0.590	0.446	0.511	0.084	−0.621	0.527	0.525	−0.140
*Cldn4*	0.758 *	0.922 *		0.707	0.880 *	0.876 *	−0.139	0.004	0.511	−0.634	0.854 *	0.311	−0.153	−0.763 *	0.202	0.658	−0.412
*Cldn5*	0.495	0.466	0.707		0.942 *	−0.434	−0.660	−0.546	0.532	−0.386	0.879 *	0.381	−0.707	−0.818 *	−0.395	0.949 *	−0.817 *
*Cxcl10*	0.560	0.738	0.880 *	0.942 *		−0.464	−0.542	−0.416	0.511	−0.509	0.943 *	0.460	−0.449	−0.879 *	−0.255	0.914 *	−0.690
*Cxcl9*	0.422	0.609	0.876 *	−0.434	−0.464		0.894 *	0.778 *	−0.144	−0.621	−0.542	0.197	0.204	0.390	0.932 *	−0.468	0.050
*Muc2*	0.096	0.234	−0.139	−0.660	−0.542	0.894 *		0.838 *	−0.283	−0.098	−0.550	0.063	0.422	0.288	0.938 *	−0.722	0.288
*Muc3*	−0.132	0.408	0.004	−0.546	−0.416	0.778 *	0.838 *		−0.540	−0.190	−0.534	0.264	0.792 *	0.545	0.802 *	−0.618	0.646
*Muc13*	0.867 *	0.213	0.511	0.532	0.511	−0.144	−0.283	−0.540		−0.050	0.695 *	−0.127	−0.711 *	−0.747 *	−0.055	0.425	−0.503
*Nos2*	−0.335	−0.590	−0.634	−0.386	−0.509	−0.621	−0.098	−0.190	−0.050		−0.431	−0.295	0.125	0.092	−0.318	−0.541	0.536
*Nt5e*	0.650	0.446	0.854 *	0.879 *	0.943 *	−0.542	−0.550	−0.534	0.695 *	−0.431		0.023	−0.510	−0.808 *	−0.277	0.838 *	−0.592
*Tff1*	0.101	0.511	0.311	0.381	0.460	0.197	0.063	0.264	−0.127	−0.295	0.023		−0.167	0.170	0.208	0.514	−0.113
*Tff2*	−0.556	0.084	−0.153	−0.707	−0.449	0.204	0.422	0.792 *	−0.711 *	0.125	−0.510	−0.167		0.562	0.276	−0.636	0.842 *
*Tff3*	−0.706	−0.621	−0.763 *	−0.818 *	−0.879 *	0.390	0.288	0.545	−0.747 *	0.092	−0.808 *	0.170	0.562		0.071	−0.708	0.627
*Tjp1*	0.372	0.527	0.202	−0.395	−0.255	0.932 *	0.938 *	0.802 *	−0.055	−0.318	−0.277	0.208	0.276	0.071		−0.494	0.155
*Tnf*	0.330	0.525	0.658	0.949 *	0.914 *	−0.468	−0.722	−0.618	0.425	−0.541	0.838 *	0.514	−0.636	−0.708	−0.494		−0.821 *
*Tlr4*	−0.474	−0.140	−0.412	−0.817 *	−0.690	0.050	0.288	0.646	−0.503	0.536	−0.592	−0.113	0.842 *	0.627	0.155	−0.821 *	

**Table 3:** Correlations between parameters measured in intestinal organoids after the addition of feces from obese asthmatic children. A higher-stringency Pearson correlation coefficient, calculated with all parameters in the study, is shown. *: *p* < 0.05.

	*Cd14*	*Cldn1*	*Cldn4*	*Cldn5*	*Cxcl10*	*Cxcl9*	*Muc2*	*Muc3*	*Muc13*	*Nos2*	*Nt5e*	*Tff1*	*Tff2*	*Tff3*	*Tjp1*	*Tnf*	*Tlr4*
*Cd14*		0.284	0.260	0.446	0.177	0.197	0.615 *	0.672 *	0.509 *	0.085	0.177	−0.212	−0.191	−0.151	0.312	0.474	0.001
*Cldn1*	0.284		0.661 *	0.658 *	0.638 *	0.845 *	0.007	0.452	0.243	0.248	0.810 *	−0.054	−0.197	0.071	0.603 *	0.432	0.540
*Cldn4*	0.260	0.661 *		0.359	0.733 *	0.587 *	0.120	0.539 *	0.486 *	0.020	0.492 *	0.443	0.374	0.498 *	0.504 *	0.383	0.053
*Cldn5*	0.446	0.658 *	0.359		0.789 *	0.453	0.191	0.743 *	0.156	0.576	0.370	0.108	−0.219	0.045	0.028	0.737 *	0.192
*Cxcl10*	0.177	0.638 *	0.733 *	0.789 *		0.549 *	0.321	0.703 *	0.500 *	0.330	0.436	0.475	0.475 *	0.678 *	0.184	0.902 *	−0.075
*Cxcl9*	0.197	0.845 *	0.587 *	0.453	0.549 *		−0.065	0.391	0.395	0.091	0.770 *	−0.113	0.220	0.306	0.570	0.351	0.357
*Muc2*	0.615 *	0.007	0.120	0.191	0.321	−0.065		0.522 *	0.340	−0.035	0.059	−0.097	−0.003	0.074	0.104	0.718 *	−0.186
*Muc3*	0.672 *	0.452	0.539 *	0.743 *	0.703 *	0.391	0.522 *		0.661 *	0.432	0.398	0.395	0.365	0.401	0.097	0.770 *	0.024
*Muc13*	0.509 *	0.243	0.486 *	0.156	0.500 *	0.395	0.340	0.661 *		0.263	0.573 *	0.222	0.093	0.616 *	0.636 *	0.329	0.360
*Nos2*	0.085	0.248	0.020	0.576 *	0.330	0.091	−0.035	0.432	0.263		0.022	0.206	−0.030	0.370	−0.005	0.149	0.576 *
*Nt5e*	0.177	0.810 *	0.492 *	0.370	0.436	0.770 *	0.059	0.398	0.573 *	0.022		0.102	0.086	0.398	0.731 *	0.357	0.413
*Tff1*	−0.212	−0.054	0.443	0.108	0.475	−0.113	−0.097	0.395	0.222	0.206	0.102		0.675 *	0.694 *	−0.060	0.386	−0.012
*Tff2*	−0.191	−0.197	0.374	−0.219	0.475 *	0.220	−0.003	0.365	0.093	−0.030	0.086	0.675 *		0.450	−0.146	0.549 *	−0.064
*Tff3*	−0.151	0.071	0.498 *	0.045	0.678 *	0.306	0.074	0.401	0.616 *	0.370	0.398	0.694 *	0.450		0.242	0.357	0.277
*Tjp1*	0.312	0.603 *	0.504 *	0.028	0.184	0.570 *	0.104	0.097	0.636 *	−0.005	0.731 *	−0.060	−0.146	0.242		−0.043	0.578 *
*Tnf*	0.474	0.432	0.383	0.737 *	0.902 *	0.351	0.718 *	0.770 *	0.329	0.149	0.357	0.386	0.549 *	0.357	−0.043		−0.213
*Tlr4*	0.001	0.540	0.053	0.192	−0.075	0.357	−0.186	0.024	0.360	0.576	0.413	−0.012	−0.064	0.277	0.578 *	−0.213	

**Table 4:** Primers used for RT-qPCR analysis purchased from Sigma Aldrich (San Luis, MO, USA. EE. UU).

Gene	Forward Sequence	Reverse Sequence
*18S*	ACACGGACAGGATTGACAGATTG	GCCAGAGTCTCGTTCGTTATCG
*Hprt*	AGGGATTTGAATCACGTTTG	TTTACTGGCAACATCAACAG
*Tnf*	CGTGGAACTGGCAGAAGAGG	CAGGAATGAGAAGAGGCTGAGAC
*Nos2*	CATCAACCAGTATTATGGCTC	TTTCCTTTGTTACAGCTTCC
*Cd14*	GAATCTACCGACCATGGAG	AAGTTGCAGGAACAACTTTC
*Nt5e*	CTATGAGCCTCTTGAAATGG	CTGATATCTTGATCACCAGAG
*Cxcl9*	GAGGAACCCTAGTGATAAGG	GTTTGATCTCCGTTCTTCAG
*Cxcl10*	AAAAAGGTCTAAAAGGGCTC	AATTAGGACTAGCCATCCAC
*Muc13*	CTTTTTGTGTTGCTGTAACG	CAAATGGACACTCTTCACAC
*Tff2*	CTGTGGAAGATTGTCACTAC	AGATTGATGAAGTCTGGTTG
*Tff3*	CCTGGTTGCTGGGTCCTCTG	GCCACGGTTGTTACACTGCTC
*Reg3g*	CAGAGGTGGATGGGAGTGGAG	CACAGTGATTGCCTGAGGAAGAG
*Claudin1*	CAATGCCAGGTATGAATTTG	TCACACATAGTCTTTCCCAC
*Claudin4*	GACTGTGCAAAGTTACTAGC	ACCAGCAATTTGGATGTAAG
*Claudin5*	AACAGTTCCTACTGAGATCC	CTTTTTAACACGTCCCTCTG
*Tjp1*	GGGGCCTACACTGATCAAGA	TGGAGATGAGGCTTCTGCTT
*Cnr1*	*AATGCCATTTAGGTGTTCTG*	*ATAGGTCTTAGAACCAACCC*
*Tlr4*	*GATCAGAAACTCAGCAAAGTC*	*TGTTTCAATTTCACACCTGG*
*Muc 2*	CCCAGAAGGGACTGTGTATG	TTGTGTTCGCTCTTGGTCAG
*Muc 3*	AAAGATTACCTCCCATCTCC	TAAAACTAAGCATGCCCTTG
*Muc 4*	*GGATTCCTTCTACGTTACAG*	*GTAGAGAAATCAGCATCAG*
*mt-Rnr1*	GACACCTTGCCTAGCCACAC	TGGCTGGCACGAAATTTACC
*mt-Rnr2*	ACTAGCATGAACGGCTAAACG	AAGCTCCATAGGGTCTTCTCG

**Table 5:** Primers used for RT-qPCR purchased from Bio-Rad (Hercules, California, EE. UU).

Gene	UniqueAssay ID
*mt-Atp6*	qMmuCED0051651
*mt-Co1*	qMmuCED0061464
*mt-Co2*	qMmuCED0027
*mt-Cyb*	qMmuCED0001514
*mtNd6*	qMmuCED0061740
*mtNd1*	qMmuCED0003246

## Data Availability

Data are available on request.
